# Increase of cells expressing PD‐1 and PD‐L1 and enhancement of IFN‐γ production via PD‐1/PD‐L1 blockade in bovine mycoplasmosis

**DOI:** 10.1002/iid3.173

**Published:** 2017-05-24

**Authors:** Shinya Goto, Satoru Konnai, Tomohiro Okagawa, Asami Nishimori, Naoya Maekawa, Satoshi Gondaira, Hidetoshi Higuchi, Masateru Koiwa, Motoshi Tajima, Junko Kohara, Satoshi Ogasawara, Yukinari Kato, Yasuhiko Suzuki, Shiro Murata, Kazuhiko Ohashi

**Affiliations:** ^1^ Department of Disease Control, Graduate School of Veterinary Medicine Hokkaido University Sapporo Japan; ^2^ School of Veterinary Medicine Rakuno Gakuen University Ebetsu Japan; ^3^ Hokkaido Research Organization, Agriculture Research Department Animal Research Center Shintoku Japan; ^4^ Department of Regional Innovation Tohoku University Graduate School of Medicine Sendai Japan; ^5^ Department of Antibody Drug Development Tohoku University Graduate School of Medicine Sendai Japan; ^6^ Division of Bioresources, Research Center for Zoonosis Hokkaido University Sapporo Japan; ^7^ Global Station for Zoonosis Control, Global Institution for Collaborative Research and Education (GI‐CoRE) Hokkaido University Sapporo Japan

**Keywords:** Bovine mycoplasmosis, IFN‐γ, immunosuppression, PD‐1, PD‐L1

## Abstract

**Introduction:**

Bovine mycoplasma, chiefly *Mycoplasma bovis*, is a pathogen that causes pneumonia, mastitis, arthritis, and otitis media in cattle. This pathogen exerts immunosuppressive effects, such as the inhibition of interferon production. However, the mechanisms involved in bovine mycoplasmosis have not been fully elucidated. In this study, we investigated the role of the programmed death‐1 (PD‐1)/programmed death‐ligand 1 (PD‐L1) pathway in immunosuppression in bovine mycoplasmosis.

**Methods:**

In the initial experiments, we used enzyme‐linked immunosorbent assay to measure interferon‐γ (IFN‐γ) from peripheral blood mononuclear cells (PBMCs) isolated from cattle with mycoplasmosis.

**Results:**

Expectedly, IFN‐γ production significantly decreased in cattle with mycoplasmosis compared with that in clinically healthy cattle. Concomitantly, flow cytometric analysis revealed that the proportions of PD‐1^+^CD4^+^ and PD‐L1^+^CD14^+^ cells significantly increased in peripheral blood of the infected cattle. Interestingly, the number of PD‐1^+^CD4^+^ and PD‐1^+^CD8^+^ T cells were negatively correlated with IFN‐γ production from PBMCs in bovine mycoplasmosis. Additionally, blockade of the PD‐1/PD‐L1 pathway in vitro by anti‐bovine PD‐1‐ and anti‐bovine PD‐L1 antibodies significantly upregulated the production of IFN‐γ from anti‐mycoplasma‐specific cells.

**Conclusions:**

These results suggest that the PD‐1/PD‐L1 pathway could be involved in immune exhaustion of bovine mycoplasma‐specific T cells. In conclusion, our study opens up a new perspective in the therapeutic strategy for bovine mycoplasmosis by targeting the immunoinhibitory receptor pathways.

## Introduction

Bovine mycoplasmosis is a disease of cattle characterized by chronic pneumonia, therapy‐resistant mastitis, otitis media, and arthritis 1, 2, 3. Many mycoplasmas such as *Mycoplasma bovis*, *Mycoplasma mycoides* subsp. *mycoides* biotype Small Colony (MmmSC), *Mycoplasma bovigenitalium*, *Mycoplasma californicum*, are known to have pathogenicity against cattle 2, 3, 4. In recent years, outbreaks of such diseases, especially of those caused by *M. bovis*, have been frequently reported worldwide, including in Japan, and have had significant economic impacts on the cattle industry 2, 3, 5, 6. Once clinical symptoms develop, it is difficult to completely cure the disease, resulting in calf mortality, weight loss in surviving calves, and decreased milk production in dairy cows 1, 2, 3. In addition, there are no effective vaccines against bovine mycoplasmas except for MmmSC; therefore, it is difficult to control this infection 4. Thus, there is a critical need to develop improved preventative and therapeutic strategies for this disease.

This pathogen exerts several immunosuppressive effects in vitro, such as inhibition of bovine peripheral blood mononuclear cell (PBMC) proliferation, induction of bovine lymphocyte apoptosis, and delay of bovine monocyte apoptosis, along with suppression of interferon (IFN)‐γ and tumor necrosis factor (TNF)‐α production 7, 8, 9. These characteristics could be associated with disease progression of bovine mycoplasmosis. However, immunosuppression mechanisms involved in bovine mycoplasmosis have not yet been fully elucidated.

Dysfunction of antigen‐specific T cells called T‐cell exhaustion has been shown to be involved in immunosuppression during various chronic infections 10, 11, 12. Exhausted T cells lose their effector functions and are phenotypically characterized by the surface expression of immunoinhibitory receptors such as programmed death 1 (PD‐1) 10. PD‐1 is expressed on the surface of activated lymphocytes, and PD‐ligand 1 (PD‐L1), a ligand for PD‐1, is expressed widely on several cells including activated lymphocytes, dendritic cells, and monocytes. Binding of PD‐1 to PD‐L1 transmits inhibitory signals into cells, subsequently exhausts T cell signaling, and induces immunosuppression 10. Recent studies have indicated the PD‐1/PD‐L1 interactions have been closely associated with the inhibition of chronically activated pathogen‐specific T cells and induction of T‐cell exhaustion 11, 12, 13, 14.

Previous studies on chronic bovine infections, such as bovine leukemia virus infection, Johne's disease, and bovine anaplasmosis, revealed that the upregulation of bovine PD‐1 and other immunoinhibitory receptors on T cells was closely associated with the exhaustion of T‐cell responses and disease progression 15, 16, 17, 18, 19, 20. Moreover, blockade of the PD‐1/PD‐L1 pathway reactivated T‐cell functions such as proliferation and cytokine production in vitro 15, 17, 18, 19, 20. However, the expression of PD‐1/PD‐L1 and their functions in bovine mycoplasmosis have not been investigated. In the present study, we determined whether the PD‐1/PD‐L1 pathway downregulates *M. bovis*‐specific T‐cell responses during bovine mycoplasmosis. In these analyses, PD‐1/PD‐L1‐expressing immunocytes increased in the peripheral blood of bovine mycoplasma‐infected cattle. Increased PD‐1‐expressing T cells were associated with decreased IFN‐γ production from bovine PBMCs. Furthermore, in vitro blockade using anti‐PD‐1 monoclonal antibodies (mAbs) or anti‐PD‐L1 mAbs restored IFN‐γ production from PBMCs stimulated by *M. bovis* antigens. The present data indicate that exhausted T cells induced by PD‐1/PD‐L1 interactions could contribute to the immunosuppression of bovine mycoplasmosis and suggest that PD‐1 and PD‐L1 are molecular targets for the control of bovine mycoplasma‐specific T‐cell responses.

## Materials and Methods

### Bovine samples and cell preparation

Peripheral blood samples of cattle with clinical symptoms were obtained from adult Holstein‐breed cattle in Hokkaido, Japan. Cattle infected with *M. bovis* were clinically and microbiologically diagnosed at the Animal Medical Center, School of Veterinary Medicine, Rakuno Gakuen University, in 2016. The symptoms of the infected cattle were pneumonia, arthritis, and otitis media. *M. bovis* infection was confirmed with PCR by using clinical samples as previously described 21. Control blood samples were collected from clinically healthy cattle with no history of mycoplasmosis maintained at the Field Science Center for Northern Biosphere, Hokkaido University, or at dairy farms in Hokkaido. All of control cattle were serologically negative for *M. bovis* infection by using enzyme‐linked immunosorbent assay (ELISA). Briefly, flat‐bottom 96‐well plates (Thermo Fisher Scientific, Waltham, MA) were coated with 100 µl of solubilized *M. bovis* (PG45, ATCC 25523, 50 µg/mL in carbonate buffer) as the target antigen at 37°C for 17 h. After the plates were four times washed with a wash solution (PBS with 0.1%Tween20), 100 µl of serum samples were added on each plate. After 37°C for 1 h incubation, plates were three times washed with a TSB‐T (PBS with 50 mM Tris, 0.1%BSA and 0.05%Tween20) and incubated with a skim milk (Wako, Osaka, Japan) as protein blocker for 2 h at 37°C. After three times washed with a TSB‐T, protein‐G conjugated horseradish peroxidase (Rockland, ME) were added to the wells and incubated 1h at 37°C. After three times washed with a TSB‐T, 3‐ethylbenzothiazolin‐6‐sulfonic Acid (ABTS; Sera care, USA) were added to the wells and measured 415 nm optical density (OD) using plate reader (iMark™ Microplate Absorbance Reader, Bio‐Rad, Hercules, CA). Bovine PBMCs were purified from blood samples with density gradient centrifugation on Percoll (GE Healthcare, Buckinghamshire, UK).

### IFN‐γ assay

To examine the decrease in IFN‐γ production in bovine mycoplasmosis, purified PBMCs were incubated with anti‐bovine CD3 antibody (2 µg/mL; MM1A, Washington State University Monoclonal Antibody Center, Pullman, WA) and anti‐bovine CD28 antibody (2 µg/mL; CC220, Bio‐Rad) in RPMI 1640 medium (Sigma–Aldrich, St. Louis, MO) containing 10% fetal calf serum (FCS; Thermo Fisher Scientific) and 100 IU/mL penicillin, 100 µg/mL streptomycin, and 2 mM L‐glutamine (Thermo Fisher Scientific) at 37°C under 5% CO_2_ for 5 days. Collected culture supernatants were assayed for IFN‐γ using an ELISA kit (Mabtech, Nacka Strand, Sweden), in accordance with the manufacturer's instructions. Data are presented as means of duplicate samples.

### Flow cytometric analysis of PD‐1 and PD‐L1

To examine the expression levels of PD‐1 and PD‐L1 in bovine mycoplasmosis, purified PBMCs were analyzed with flow cytometry. PBMCs were isolated from blood and incubated in PBS containing 10% goat serum (Sigma–Aldrich) at room temperature for 15 min to prevent nonspecific reactions. Cells were then washed and stained with anti‐ bovine PD‐1 mAb (5D2, rat IgG2a; 15) or rat IgG2a isotype control (R35‐95, BD Biosciences, San Jose, CA) for 30 min at room temperature. After being washed with PBS containing 1% bovine serum albumin (BSA) (Sigma–Aldrich), cells were stained with FITC‐conjugated anti‐CD4 mAb (CC8, Bio‐Rad), R‐PE‐conjugated anti‐CD8 mAb (CC63, Bio‐Rad), PerCp/Cy5.5‐conjugated anti‐CD3 mAb (MM1A, Washington State University Monoclonal Antibody Center), PE/Cy7‐conjugated anti‐IgM mAb (IL‐A30, Bio‐Rad), and APC‐conjugated anti‐rat immunoglobulin antibody (Southern Biotech, Birmingham, AL) antibody conjugates for 30 min at room temperature. Before the staining, MM1A and IL‐A30 were conjugated with PerCp/Cy5.5 and PE/Cy7, respectively, using Lightning‐Link conjugation kits (Innova Biosciences, Cambridge, UK). Stained cells were then washed and immediately analyzed using FACS Verse (BD Biosciences) and FCS Express 4 (De Novo Software, Glendale, CA). To detect PD‐L1‐expressing cells, PBMCs were blocked with PBS containing 10% goat serum and washed and stained with anti‐bovine PD‐L1 mAb (4G12, rat IgG2a; 20) or rat IgG2a isotype control (R35‐95, BD Biosciences) in the presence of anti‐CD11b mAb (CC126, mouse IgG2b, Bio‐Rad) for 30 min at room temperature. After washing with PBS containing 1% BSA, cells were stained with APC‐Cy7‐conjugated anti‐CD14 mAb (CAM36A: Washington State University Monoclonal Antibody Center) using the Lightning‐Link APC‐Cy7 tandem conjugation kit (Innova Biosciences), APC‐conjugated anti‐rat immunoglobulin antibody (Southern Biotech), and FITC‐conjugated anti‐mouse IgG2b antibody (Beckman Coulter, Fullerton, CA) antibody conjugates for 30 min at room temperature. Cells were then washed and immediately analyzed using FACS Verse (BD Biosciences) and FCS Express 4 (De Novo Software) as above.

### Blockade assay

To investigate the influence of PD‐1/PD‐L1 blockade, PBMCs were incubated with 10 μg/mL anti‐bovine PD‐1 mAb (5D2; 15) or anti‐bovine PD‐L1 mAb (4G12; 20) in the presence of 1.5 µg/mL heat‐killed *M. bovis* (PG45, ATCC 25523). Rat IgG (Sigma–Aldrich) was used as a control antibody. All antibodies used in blockade assay were suspended in endotoxin‐free sterile PBS (Wako) without azide. PBMC cultures were grown in 96‐well plates (Corning, Inc., Corning, NY) at 37°C with 5% CO_2_ for 5 days. Subsequently, culture supernatants were harvested from individual wells and were tested for a bovine IFN‐γ ELISA (Mabtech) as described above.

### Statistics

Differences were identified using Wilcoxon's matched pairs test and Spearman's rank test. Steel–dwass test was used to compare the expression of PD‐1 and PD‐L1 among types of mycoplasmosis with different clinical symptoms. All statistical tests were performed using the statistical analysis program MEPHAS (http://www.gen-info.osaka-u.ac.jp/MEPHAS/). *P* values of <0.05 were considered significant.

## Results

### Reduction in the IFN‐γ production in bovine mycoplasmosis

**Figure 1:**
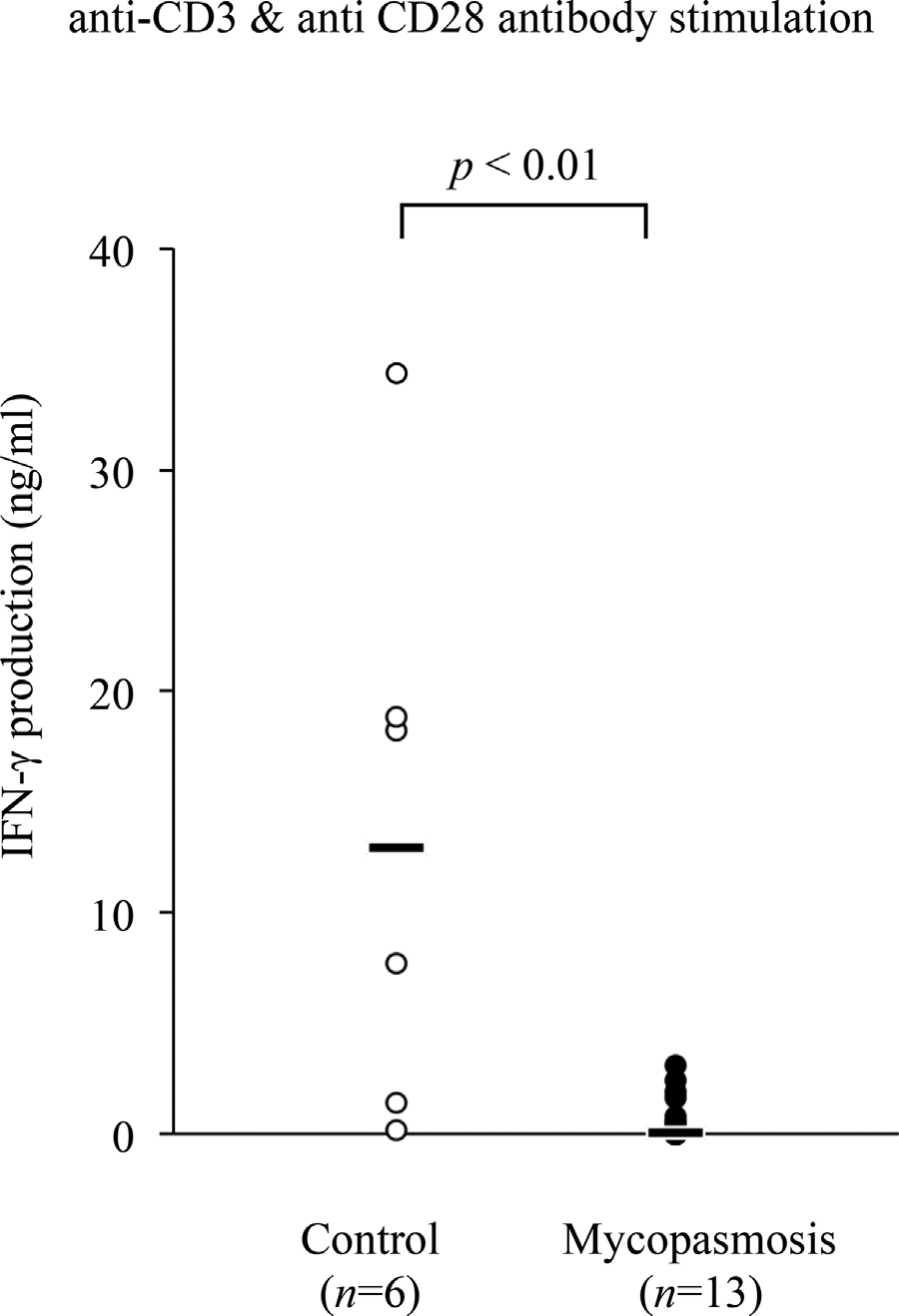
IFN‐γ production in bovine mycoplasmosis. IFN‐γ production was determined in supernatant of the PBMCs culture from mycoplasmosis‐afflicted and healthy control Holstein cattle by ELISA assay. PBMCs were incubated with the combination of anti‐bovine CD3 and anti‐bovine CD28 antibodies. Each line indicates the median production in each group.

The median IFN‐γ in supernatant of cultivated PBMCs that were stimulated with anti‐CD3 and anti‐CD28 antibodies in the cattle with mycoplasmosis was significantly lower than that in the healthy cattle (*P* < 0.01) (Fig. 1). No significant differences were observed in IFN‐γ production among the different clinical symptoms (pneumonia, arthritis, and otitis media) in the *M. bovis*‐infected cattle (data not shown).

### Upregulation of PD‐1/PD‐L1 expression in bovine mycoplasmosis

**Figure 2:**
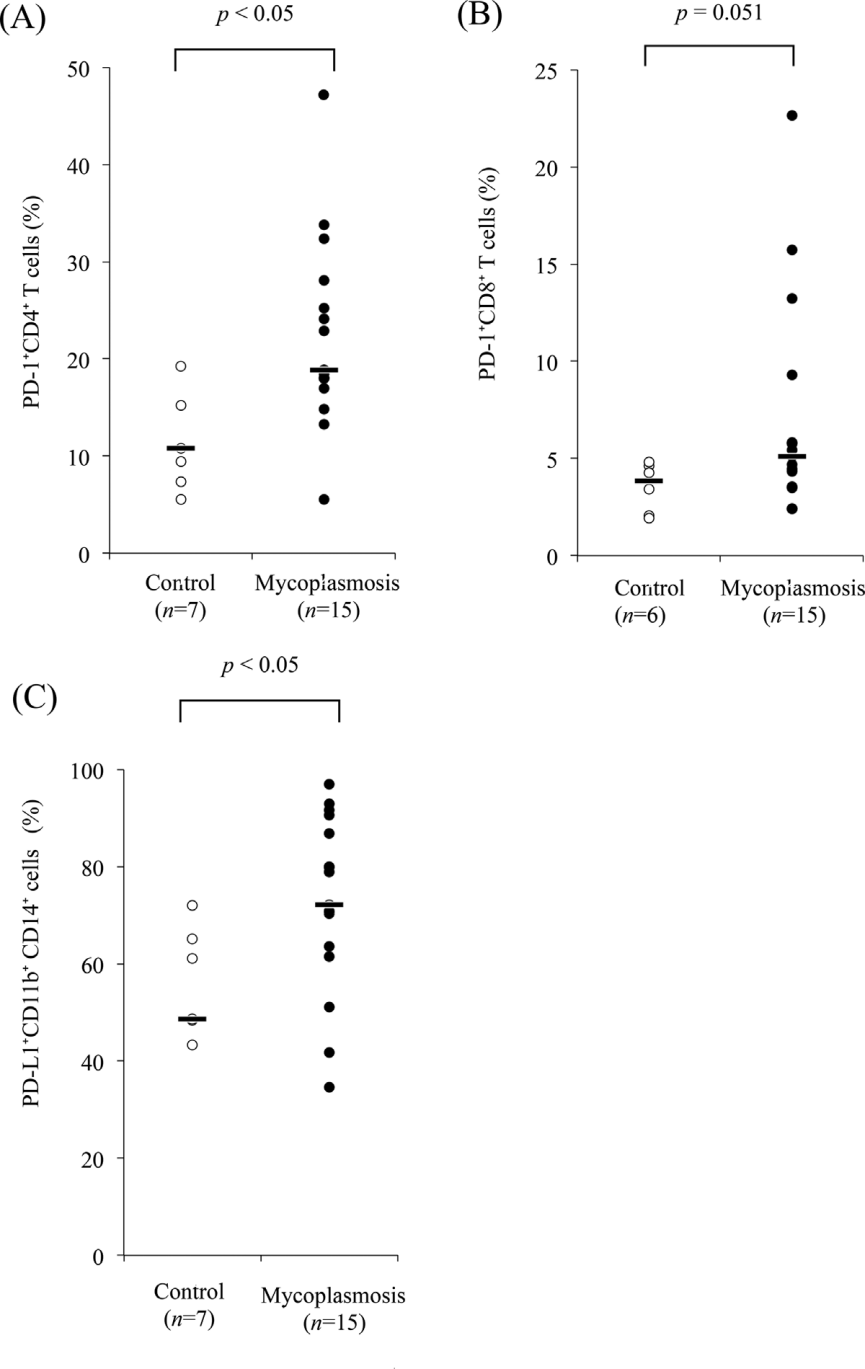
Expression analysis of PD‐1 and PD‐L1 in bovine mycoplasmosis. Flow cytometric analysis of the PD‐1 expression on CD4^+^ T cells (A), CD8^+^ T cells (B), and PD‐L1 expression on CD11b^+^CD14^+^ monocytes (C) in PBMCs from cattle with mycoplasmosis and healthy control cattle. Each line indicates the median percentage in each group.

**Figure 3:**
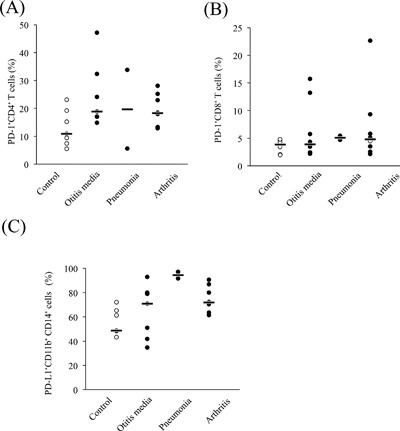
Comparative analysis of PD‐1 and PD‐L1 expression among bovine mycoplasmosis cases with different symptoms. PBMCs from *M. bovis*‐infected cattle with otitis media (*n* = 7), pneumonia (*n* = 2), and arthritis (*n* = 8) were analyzed.

**Figure 4:**
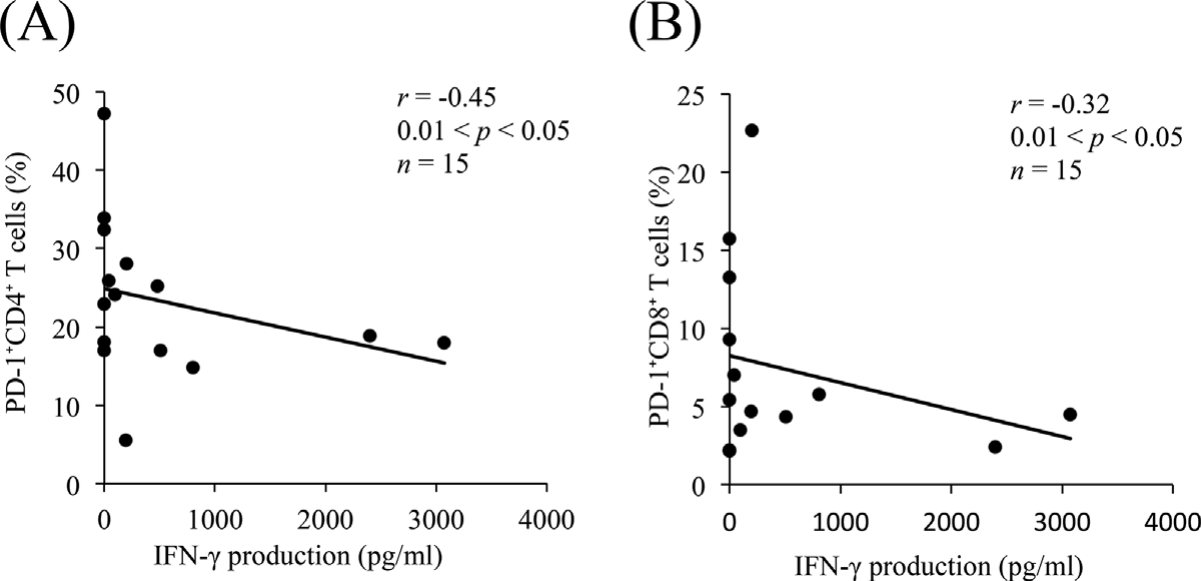
Negative correlation between the proportion of PD‐1^+^ T cells and IFN‐γ production. IFN‐γ production in cattle with mycoplasmosis corresponds to that in Figure 1A (*n* = 15). Correlation statistics were analyzed using Spearman's correlation.

To investigate PD‐1 expression in bovine mycoplasmosis, we examined the proportions of PD‐1^+^ cells among PBMCs freshly isolated from *M. bovis*‐infected cattle with pneumonia, arthritis, or otitis media. The median proportion of PD‐1^+^CD4^+^ (Fig. 2A) among PBMCs isolated from cattle with mycoplasmosis were higher than those from healthy control cattle. In addition to this, there was approaching significance difference in proportion of PD‐1^+^CD8^+^ T cells between *M. bovis*‐infected cattle and control cattle (*P* = 0.051) (Fig. 2B). Similarly, the median proportion of PD‐L1^+^CD11b^+^CD14^+^ cells was higher in cattle with mycoplasmosis than in control cattle (*P* < 0.05) (Fig. 2C). No significant differences were observed in PD‐1 and PD‐L1 expressions among the types of mycoplasmosis with different clinical symptoms in the *M. bovis*‐infected cattle, although all of the median proportions of PD‐1^+^CD4^+^ (Fig. 3A), PD‐1^+^CD8^+^ (Fig. 3B), and PD‐L1^+^CD11b^+^CD14^+^ cells (Fig. 3C) were higher than those of healthy control cattle. Interestingly, the increases in the proportion of PD‐1^+^CD4^+^ and PD‐1^+^CD8^+^ T cells negatively correlated with IFN‐γ production from PBMCs in infected cattle (Fig. 4A and B).

### Reactivation of IFN‐γ production by PD‐1/PD‐L1 blockade

**Figure 5:**
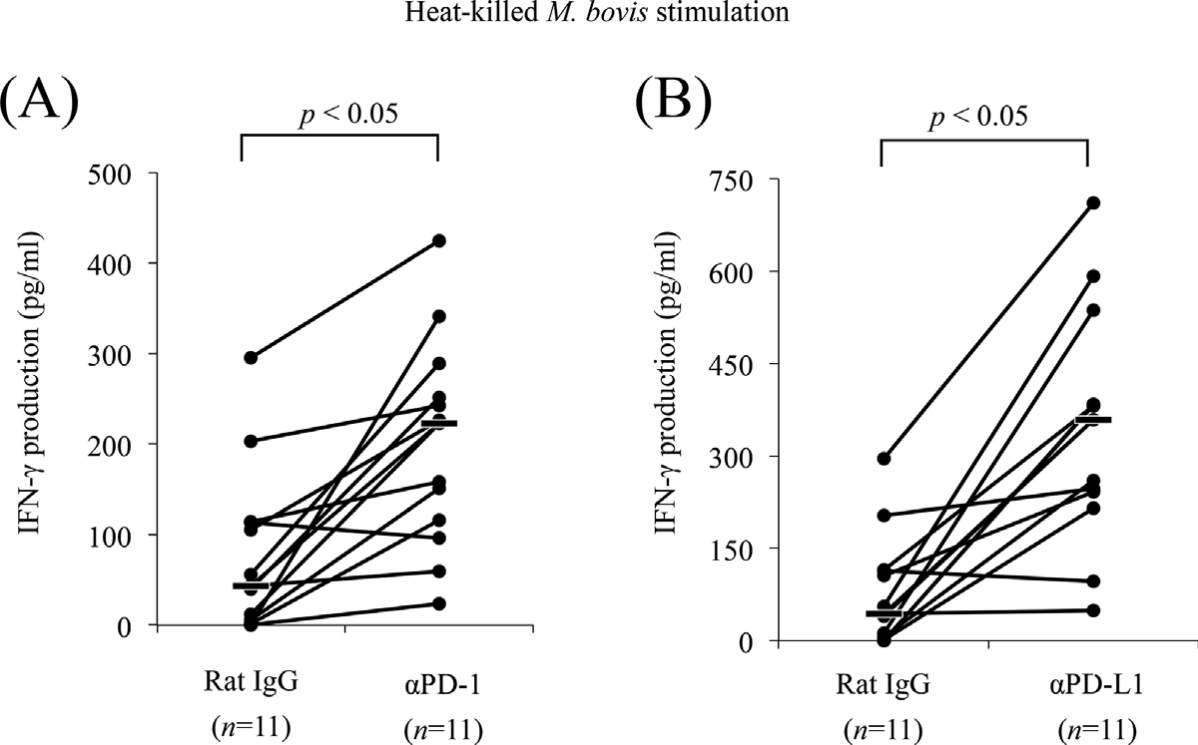
Enhancement of IFN‐γ production by anti‐PD‐1 and anti‐PD‐L1 mAbs in PBMCs from cattle with mycoplasmosis. PBMCs were cultivated with rat IgG control, anti‐PD‐1 mAb (A: 20 µg/mL), or anti‐PD‐L1 mAb (B: 20 µg/mL) in the presence of heat‐killed *M. bovis*. IFN‐γ production was measured by ELISA assay. Statistical comparisons between rat IgG control and blocking mAb were performed using Wilcoxon's matched‐pairs test.

To test the effect of the PD‐1/PD‐L1 blockade on *M. bovis*‐specific immune response, PBMCs from *M. bovis*‐infected cattle were cultivated in the presence of heat‐killed *M. bovis* together with anti‐PD‐1, anti‐PD‐L1, or an isotype control antibody. IFN‐γ production was significantly augmented in cells treated with the anti‐PD‐1 antibody compared with those treated with the control antibody (Fig. 5A). Similarly, the blockade by anti‐PD‐L1 antibody also enhanced IFN‐γ production from PBMCs stimulated with *M. bovis* (Fig. 5B).

## Discussion

During chronic infection, pathogens evade host immune responses and persist after the effector phase 22, 23, leading to persistent antigen stimulation and progressive T‐cell dysfunction known as T‐cell exhaustion 10. Exhausted T cells are controlled by the immunoinhibitory receptor PD‐1 on the cell surface via T‐cell inhibitory signals that follow crosslinking to the ligand PD‐L1 13. Previous studies revealed that the expression of PD‐1 was upregulated on exhausted T cells in various bovine chronic infections such as bovine leukemia virus infection, Johne's disease, and bovine anaplasmosis 15, 16, 17. T cells expressing these receptors show low effector functions and allow chronic pathogens to establish persistent infection. Therefore, T‐cell exhaustion is regarded as the common mechanism of immune evasion in chronic infections.

In bovine mycoplasmosis, it is well‐known that pathogens, chiefly *M. bovis*, exert immunosuppressive effects in vitro 7, 8, 9. Because of these characteristics, it is suspected that this disease develops to chronic progression and, especially during lung infection, allows to establish co‐infection with other bacteria and other pathogens such as viruses 2. However, the mechanisms involved in bovine mycoplasmosis have not yet been fully elucidated. In this study, we investigated the role of the PD‐1/PD‐L1 pathway in immunosuppression in cattle infected with bovine mycoplasma.

IFN‐γ is a Th1 cytokine mediating a cellular immune response by promoting the activation and proliferation of macrophages. In contagious bovine pleuropneumonia caused by MmmSC, a positive correlation between the number of MmmSC‐specific IFN‐γ‐secreting CD4^+^ T cells and the recovery from the disease was identified 24, 25. Therefore, IFN‐γ production might be important for protection against bovine mycoplasmosis. In the current study, *M. bovis*‐specific IFN‐γ production from PBMCs decreased in cattle with bovine mycoplasmosis compared with that healthy control cattle. This suggested that the immune response against *M. bovis* might not be effective during bovine mycoplasmosis.

In the present study, PD‐L1 expression on CD14^+^CD11b^+^ monocytes and PD‐1 expression on CD4^+^ T cells was found to be upregulated in bovine mycoplasmosis. We also compared PD‐1 or PD‐L1 expression among different conditions of bovine mycoplasmosis. There were no significant differences among the different conditions, but the proportions of PD‐1‐ or PD‐L1‐expressing cells in all conditions increased compared with those in healthy cattle. Moreover, lower proportions of circulating PD‐1^+^ cells strongly correlated with increased IFN‐γ levels. This indicates that decreased levels of IFN‐γ during the progression of bovine mycoplasmosis could be due to the increased number of PD‐1^+^ cells. These results suggest that the PD‐1/PD‐L1 pathway could be involved in immune exhaustion of bovine mycoplasma‐specific T cells.

In chronic infections, the expression of PD‐1 is induced by continuous antigen presentation and T‐cell receptor stimulation 10, 13. Thus, PD‐1 was hypothesized to be upregulated on *M. bovis* antigen‐specific T cells, causing effector function loss in these cells.

The mechanism of PD‐L1 upregulation during bovine mycoplasmosis remains unknown. In HIV models, the cytokine microenvironment was proposed as one of the mechanisms by which PD‐L1 expression is elevated 26. This hypothesis might contribute to understanding the mechanism of PD‐L1 upregulation during mycoplasmosis, considering the changes in the cytokine microenvironment 27, 28, 29. Nevertheless, further elucidation of the mechanism underlying the elevation of PD‐L1 expression is warranted to obtain a comprehensive understanding of cell signaling pathways involved in the modulation of host immune responses.

In vitro blockade assays of PD‐1/PD‐L1 pathway in PBMCs showed that blockade with anti‐PD‐1 and anti‐PD‐L1 mAbs efficiently reactivated the *M. bovis*‐specific IFN‐γ response. It is noteworthy that the blockade of the PD‐1/PD‐L1 pathway restored anti‐mycoplasma immune functions in vitro. The results obtained in this study were consistent with those in other bovine diseases, such as bovine leukemia virus (BLV) infection, Johne's disease, and bovine anaplasmosis 15, 17, 18.

For use in humans, PD‐1/PD‐L1 pathway‐targeting biopharmaceuticals have been successively developed. Recently, we established an anti‐bovine PD‐L1 rat‐bovine chimeric antibody, and clinical study was conducted in BLV infection. Interestingly, the tested calf indicated that the proliferation of anti‐BLV‐specific CD4^+^ T cells was increased by the chimeric antibody inoculation, while BLV provirus loads were significantly reduced, clearly demonstrating that this treatment induced antivirus activities 30. The current results also suggest that blockade of the PD‐1/PD‐L1 pathway might be an effective method for controlling bovine mycoplasmosis.

Here, we examined *M. bovis*‐specific IFN‐γ production as a key response of Th1‐mediated immunity and described novel mechanisms of T‐cell exhaustion mediated by immunoinhibitory receptors in bovine mycoplasmosis. However, additional studies are required to determine multiple effects of blocking mAbs during the rejuvenation of T‐cell exhaustion. Specifically, measurements of other Th1 cytokines, such as TNF‐α, interleukin (IL)‐2, and IL‐12, and of T‐cell proliferation and cytotoxic activity may reveal further aspects of T‐cell exhaustion in bovine mycoplasmosis. The present findings may contribute to the development of novel strategies for manipulating bovine mycoplasma‐specific T‐cell responses to prevent disease progression.

## Authors’ Contributions

SiG and SK performed all of the experiments, analyzed the data, and drafted the manuscript. SK participated in the experimental design, analyzed data, and helped to draft the manuscript. TO, AN, and NM participated in some experiments and sample collection. MK, MT, and JK participated in sample collection. SaG, HH, and SM assisted with data analysis and provided overall guidance for the studies. SO, YK, YS, and KO supervised the study and reviewed the manuscript. All authors read and approved the final manuscript.

## Conflict of Interest

None declared.
